# Transformative Experiences of People Living with Dementia and Their Care Partners at Walking the Talk for Dementia 2024

**DOI:** 10.1002/alz70858_097742

**Published:** 2025-12-24

**Authors:** John Crowley, Kathleen Cahill, Sherril B Gelmon, Walter D Dawson, Jonathan Adrian Zegarra‐Valdivia, Fernando Aguzzoli Peres, Lingani Mbakile‐Mahlanza, Kate Irving

**Affiliations:** ^1^ Dementia Research Alzheimer's Ireland Team, Kilkenny, Ireland; ^2^ Alzheimer's Society of Ireland, Kilkenny, Ireland; ^3^ OHSU‐PSU School of Public Health, Portland, OR, USA; ^4^ GBHI Lived Experience Panel, Portland, OR, USA; ^5^ Global Brain Health Institute, San Francisco, CA, USA; ^6^ Oregon Health & Science University, Portland, OR, USA; ^7^ Global Brain Health Institute (GBHI), University of California San Francisco, San Francisco, CA, USA; ^8^ Universidad Señor de Sipán, Chiclayo, Peru; ^9^ Cajal Institute, Madrid, Spain; ^10^ Global Brain Health Institute, Dublin, Dublin, Ireland; ^11^ Walking the Talk for Dementia, Porto Alegre, Rio Grande do Sul, Brazil; ^12^ University of Botswana, Gaborone, Botswana; ^13^ Dublin City University, Dublin, Ireland

## Abstract

**Background:**

Twenty‐three people living with dementia and their care partners participated in Walking the Talk for Dementia (WTD) in August 2024.

**Methods:**

All the people living with dementia and care partners consented to participate. Sources of data included individual reflections (written, video or audio), and pre/post survey responses about concerns, highlights, inspiration, inclusivity, stigma, courage, impact, and self‐perceptions.

**Results:**

Both people living with dementia and their care partners reflected on the courage of participants directly affected by dementia to respond to the challenges that dementia presents in their lives.

People living with dementia described a highlight of WTD as meeting other people living with dementia, spending time together, sharing stories, and learning together how to navigate their individual pathways with the disease. A comment typical of many addressed how “*… we can make life better for one another [and] we can support one another. We can understand the challenges of [dementia] and [can] encourage other people who have [dementia].”*

Care partners expressed surprise at the empathy and camaraderie of the WTD community, and how welcomed they felt, especially when their person needed additional support to navigate the Walk or related events. They shared experiences with other care partners and ideas on how to support a person living with dementia.

A common theme across all sources of evidence was how valued people living with dementia and their care partners felt during WTD. Many were excited about possible future connections within the WTD community for support, information exchange, and friendship. While most joined WTD to advance awareness about dementia, they left feeling valued and respected, recognizing how essential they are to dementia advocacy, care, and policy. WTD was “*a fantastic experience for people to share what works and what helps – the power is in the group.”*

**Conclusions:**

WTD immerses participants in shared reflection on experiences of advocacy, caring, and research. The engagement of people living with dementia and their care partners is essential to ensure others gain understanding of their experiences. WTD is an innovative platform where vulnerability and expertise are valued, fostering deep connections in a welcoming environment.

